# Dr. Paul C. Freer

**Published:** 1912-05

**Authors:** 


Dr. Paul C. Freer, director of the U. S. scientific bureau, who was mentioned as a possible successor to Dr. Wiley, died April 17, 1912. He was born in Chicago in 1862, was gradated at Rush Medical College and took postgraduate courses at the University of Munich. He was formerly Professor of Chemistry at the University of Michigan.

